# MgONPs Can Boost Plant Growth: Evidence from Increased Seedling Growth, Morpho-Physiological Activities, and Mg Uptake in Tobacco (*Nicotiana tabacum* L.)

**DOI:** 10.3390/molecules23123375

**Published:** 2018-12-19

**Authors:** Lin Cai, Minghong Liu, Zhongwei Liu, Huikuan Yang, Xianchao Sun, Juanni Chen, Shunyu Xiang, Wei Ding

**Affiliations:** 1 College of Plant Protection, Southwest University, Chongqing 400715, China; lincai0203@163.com (L.C.); kuan320914@163.com (H.Y.); sunxianchao@163.com (X.S.) chenhuanni521@126.com (J.C.); xiangshunyu0325@163.com (S.X.); 2 Zunyi Branch Company, Guizhou Tobacco Company, Zunyi 563000, China; lmh859@163.com; 3 Guizhou Key Lab of Agro-Bioengineering, Guizhou University, Guiyang 550025, China; zwliu@gzu.edu.cn

**Keywords:** tobacco plants, Mg uptake, chlorophyll, morpho-physiology, growth stimulators

## Abstract

In this study, we documented the impact of magnesium oxide nanoparticles (MgONPs) on the various morpho-physiological changes by root irrigation in tobacco plants in the matrix media, as well as the uptake and accumulation of the NPs over a range of concentrations (50–250 μg/mL). Our results showed that the seed germination rate was not affected following exposure to MgONPs for 5 days. Enhanced plant growth together with increased peroxidase activity (39.63 U mg^−1^ protein in the 250 μg/mL MgONPs treatment, 36.63 U mg^−1^ protein in the control), superoxide dismutase activity (30.15 U mg^−1^ protein compared to 26.95 U mg^−1^ protein in the control), and chlorophyll content (the chlorophyll a and b contents in 0 and 250 μg/mL of MgONPs were 0.21, 0.12 μg/g to 1.21, 0.67 μg/g, respectively) were observed after 30 days of MgONP treatment. However, the malondialdehyde, protein, and relative water contents did not differ significantly, indicating that the NPs in the test concentrations had no phytotoxicity and even promoted plant growth. Scanning electron microscopy and paraffin section observations indicated that the MgONPs did not affect the plant tissue structures and cells. In addition, an elevated Mg content was detected in the plant tissues exposed to MgONPs, suggesting that the Mg was taken up by the tobacco roots and translocated to the shoots and leaves, which were probably the most important tools to cause an increase in the chlorophyll content and stimulate growth. In particular, compared with the controls, a substantially higher Mg content was observed in the leaves (12.93 mg/g in the MgONPs treatment, 9.30 mg/g in the control) exposed to 250 μg/mL MgONPs, especially in the lower and middle leaves. This result confirmed that the contents of plant Mg-element in the old leaves were increased by MgONPs. In summary, this study investigated increased Mg uptake and growth stimulation, as well as the induction of various positive morpho-physiological changes to tobacco plants when exposed to MgONPs. Results elucidate the promotional impact of the NPs on plant health and their implications for agricultural safety and security.

## 1. Introduction

Nanotechnology, which involves compounds with less than 100 nm in at least one dimension, is a burgeoning interdisciplinary area. Given their unique superior physicochemical properties, nanomaterials are increasingly being utilized in a range of applications in agriculture, medicine, wastewater treatment, and manufacturing. Generally offering profitability and sustainability, nanomaterials are used in agriculture (particularly in pesticides), bactericides and micronutrients, which serve to improve the quality of crops [1] and reduce hunger, child mortality and malnutrition; such situations have been preliminarily verified in densely populated countries, such as China and India [2]. However, previous studies revealed that once absorbed, the biotransformation of nanomaterials in ambient environmental media may be modified and pose a toxic threat to living organisms, or they may have contrasting effects. This technology is not completely safe or environmentally friendly, despite its numerous advantages. To date, the increased applications of nanomaterials have raised a tremendous controversy factoring in their potential hazardous effects to ecosystems and human health [3]. Therefore, considering this possibility and with the aim of supporting the sustainable development of nanomaterials, possible risks must be assessed to clarify all the relevant aspects of their use [4].

Terrestrial plants, an essential component of living organisms, are critical to maintain the ecological equilibrium, and they provide food sources and safety to humans and animals worldwide [5]. In addition, plants provide a potential pathway for human exposure to nanomaterials because of their uptake and accumulation in plant tissues [6]. To understand the environmental and ecosystemic risks of nanomaterials, the behaviour of nanomaterials towards plants requires extensive research [5]. Consequently, before the extensive promotional uses of nanotechnology are implemented, the bioavailability and behaviour of nanomaterials in a particular dose in agriculture, particularly in plant systems, must be investigated [7].

To the best of our knowledge, an array of publications has reported on the uptake and accumulation of nanomaterials focusing on various plants, including bean, wheat, tomato and watermelon, over more than a decade. However, knowledge in this area remains rudimentary. These studies revealed both the complicated and unpredictable effects of nanomaterials on plants, including positive results, adverse effects and inconsequential outcomes [1,6,8]. For example, the improvement of the crop yield through beneficial physiological responses was evident in plants sprayed to TiO_2_ nanoparticles (NPs) at 10 mg/L concentration, which can be attributed to the promotion of nitrogen photoreduction in plants [9]. By contrast, some phytotoxicity studies reported a significant metabolic alteration in plants after the 50 ppm exposure of AgNPs because the uptake of N and P from the soil system to plants was substituted by the uptake of Ag [10]. However, another study indicated no remarkable impact on seed germination after treatment with ZnO NPs [11]. To a large extent, their effects on plants vary with the species and physiological basis of the plants and NPs. In addition, there is a critical concentration of some nanomaterials up to which the growth of the plant is promoted or poisoned [7]. In these studies, the researchers generally verified that the plants treated with nanomaterials at low concentrations grow better than the control; at high concentrations, alterations in the cell morphology and structural characteristics occur in the plants that suffer toxic effects [4,6,7]. In addition, there is a strong correlation amongst nitrogen metabolism, photosynthesis, plant growth and nanomaterial concentration [12].

A vital indicator of current studies of nanomaterials serve as to determine the effects of nanomaterials on plant growth and productivity with the goal of promoting their use in agricultural production. Amongst the large numbers of different nanomaterials, magnesium oxide NPs (MgONPs), with the advantages of being nontoxic, safe and easy to obtain, functions as an outstanding bactericide against *Ralstonia solanacearum* (*R. solanacearum*) and offers substantial promise to prevent root and stem diseases [13]. These results highlight the potential possibility of MgONPs as excellent alternatives to chemical bactericides in crop protection, but sufficiently detailed research about their effects on plants remains largely unknown, especially on tobacco. Therefore, there is an urgent need to delineate the interactions between the MgONPs and plants.

In this study, we measured the impact of MgONPs delivered by root application on tobacco plants. Particular efforts were focused on how the MgONPs influenced the growth of tobacco seeds and seedlings, whether the NPs were taken up and translocated in the tobacco plant tissue and morphological changes of the plant cells. In addition, detailed physiological and biochemical response assessments, such as the activities of superoxide dismutase (SOD) and peroxidase (POD) enzymes; malondialdehyde (MDA), protein and relative water contents; and chlorophyll content, were estimated to reveal a mechanistic understanding of the pathways. To our knowledge, this study is the first to provide a detailed assessment of the effects of MgONPs on tobacco plants.

## 2. Materials and Methods

### 2.1. Materials and Characterization of Nanoparticles

The MgONPs were purchased from Sigma-Aldrich. These particles were distributed in deionized water and poured into a matrix potting medium (Pindstrup Mosebrug A/S, Shanghai, China) to conduct the plant experiment. The practical size of the particles was characterized using TEM (JEM-2100, JEOL, Tokyo, Japan) and SEM (S-570, Hitachi, Tokyo, Japan), and the particles were characterized in detail as described in our previous study [13]. The nitrogen adsorption–desorption isotherms of the MgONPs were evaluated using a Micromeritics Analyzer (ASAP 2010, Norcross, GA, USA) with nitrogen. The zeta potential of the MgONP in the matrix extraction was evaluated using a Malvern Zetasizer Nano Series (Malvern, UK). The matrix was suspended in deionized water to filtrate until the large particle residue was removed to obtain the matrix extraction. The MgONPs were suspended in deionized water to obtain the final suspension concentration of 250, 150 and 50 μg/mL and sonicated for 30 min for experimental use.

### 2.2. Seed Germination Test

The test plant “Yunyan 87” a variety of flue-cured tobacco developed through pedigree selection by crossing Yun-yan No.2 and K326, is a critical economic and agricultural plant in China. The germination experiments examined the bioavailability of the nanoparticles to the seeds [14]. First, the tobacco seeds were sterilized in 2.5% sodium hypochlorite solution for 15 min, and the seeds were washed three times with deionized water to remove the sodium hypochlorite residue. Next, the seeds were dried under static conditions at low temperature and natural conditions. The MgONPs were diluted with deionized water to a series of different concentrations (50, 150 and 250 μg/mL), and the same volume of solution was added to filter paper. The same volume of deionized water served as the control treatment. Finally, the dried seeds were transferred to Petri dishes containing the wet filter paper of different concentrations of MgONPs, including 50, 150 and 250 μg/mL, and 40 seeds were placed on each Petri dish in an artificial plant growth chamber at 25 ± 2 °C (mean ± standard deviation), a relative humidity of 85% and a light period of 14 h. Each experimental treatment was repeated three times to ensure the reproducibility of the data. The germination percentage of seeds was calculated based on the amount of radicle and plumule.

### 2.3. Tobacco Plants Growth and Greenhouse Conditions

In this study, after 5 days incubation for germination, uniform germinated tobacco seedlings were selected to transplant to a pot filled with the matrix media (Pindstrup Mosebrug A/S, Shanghai, China), which contained mixing of blonde and black peat [7]. The MgONPs were applied to the tobacco plants by exposure to the root. Specifically, the plants were drenched with 50 mL of the MgONPs suspension of different concentrations (0, 50, 150 and 250 μg/mL) by root exposure at the four-leaf-old stage in the matrix potting media for 30 days of treatment. During the experiments, there were 20 plants for each treatment, and all the trials were conducted in a randomized order. The experiments were repeated three separate times.

### 2.4. Chlorophyll Determination

The chlorophyll extraction technique was performed as previously described [7] with minor modifications. In this study, the chlorophyll content of the tobacco plants was assessed after treatment with different concentrations of the MgONPs for 30 days. Tobacco plant leaf samples of the upper, middle and lower parts were collected and rinsed with tap water, cooled and dried. Subsequently, fresh plant leaves were cut into pieces, and 1 g plant leaf samples were randomly selected to dip in 100% acetone in the dark. After 12 h of incubation, the leaf pigments were centrifuged to precipitate the debris. Finally, the extracted chlorophyll samples were measured using a UV-Vis spectrophotometer (UV–2550PC, Shimadzu-China, Suzhou, China) at a 661.2 nm wavelength for chlorophyll a and a 644.8 nm wavelength for chlorophyll b. The total chlorophyll content was assessed using the following equation:

Total Chlorophyll = chlorophyll a + chlorophyll b(1)

### 2.5. Determination of Antioxidant Enzymes, MDA, Protein and Relative Water Contents

The levels of activity of the antioxidant enzymes of tobacco were examined in this experiment, including SOD and POD. In addition, the content of an antioxidant system indicator was measured using MDA. The total protein content of the tobacco was also measured. All these indicators were measured according to the manufacturer’s instructions of the test kits (Nanjing Jiancheng Bioengineering Institute, Nanjing, China).

In addition, these samples were processed to determine their relative water content [15]. Fresh leaf disc samples (FW) (200 mg) were added to distilled water for 4 h to harvest the turgid weight (TW). The dry weight (DW) was detected after drying at 80 °C for 4 h. Measurements of the relative water content (RWC) were calculated using the following formula:

RWC (%) = [FW − DW/TW − DW] × 100(2)

All the experiments were conducted in triplicate and included a control.

### 2.6. Elemental Analysis

Previous research [16] used elemental analyses to test the mobilization and accumulation of the nanoparticles in the plants. Therefore, samples of the tobacco roots, stems and leaves were initially collected, rinsed with tap water and dried separately at 70 °C for 72 h. The following procedure was to weigh these plant dried samples, and completely digested them in the 6 mL solution (HNO_3_ and hydrogen peroxide (5:1)) for 10 h at 150 °C until the solution became clear. Next, deionization water was added to the solution to obtain a constant volume (50 mL), and centrifuged at 10,000 g for 10 min to collect the supernatant. Finally, these groups of suspensions were diluted to 100 ppb with 1% HNO_3_ to assess the Mg elemental concentrations using an inductively coupled plasma mass spectrometer (ICP-OES, Perkin Elmer, Inc., Shelton, WA, USA). The experiments were repeated three separate times.

### 2.7. Structural Observation Using Paraffin Sections

The tobacco seedlings were subjected to MgONP treatments and grown as described above. In this study, the plant samples were gently excised to keep the roots, stems and leaves of the tobacco plants intact after applying the MgONPs for 30 days. These plant samples were soaked in the FAA fixed liquid (70% ethanol:formaldehyde: acetic acid, 9:1:1), and the fixed samples were dehydrated, embedded in parafilm and sliced. Finally, the staining was conducted using fan-red dye and observed using light microscopy (Leika, Portland, OR, USA, DCF425).

### 2.8. SEM Observation

To further explore the morphological changes of the tobacco plants exposed to MgONPs, the morphology of the tobacco roots, shoots and leaves was investigated using SEM. The roots, stems and leaves were cut from the tobacco plants and gently washed with PBS solution (pH 7.4). A total of 2.5% glutaraldehyde was used to fix the plant samples for 4 h, followed by two washes with PBS to remove all the glutaraldehyde. A series of ethanol treatments (concentration from 30% to 100%) for 10 min/time was used to dehydrate the samples, which were dried to their critical point. Later, the samples were coated with a thin layer of platinum using a sputter coater device to enhance the contrast when measured using SEM. After being coated, the final samples were detected using an SEM (S-3400N, Hitachi, Japan) at room temperature.

### 2.9. Statistical Analysis

All the treatments were conducted with a minimum of three replicates, and the data were represented as the mean values ± SD (standard error). Statistically significant data are represented by an asterisk for * and ** indicating *p* < 0.05 and *p* < 0.01, respectively.

## 3. Results and Discussion

### 3.1. Materials and Characterization of the NPs

**Figure 1:**
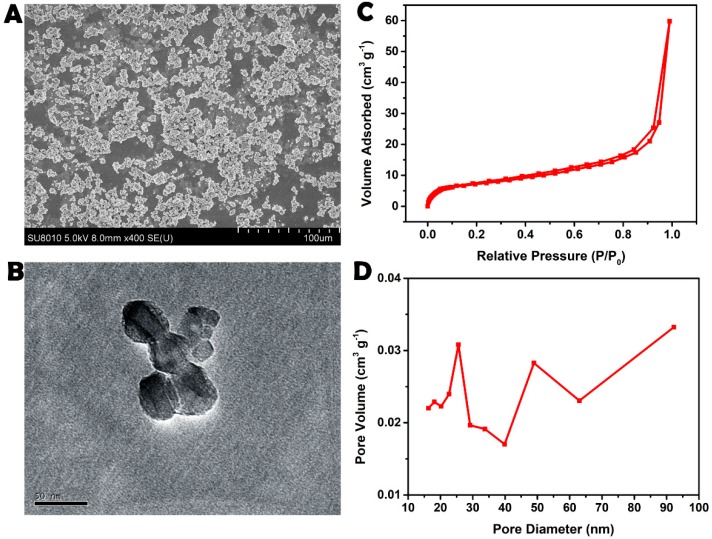
SEM image (**A**), TEM image (**B**), nitrogen adsorption-desorption isotherms (**C**) and their corresponding pore size distribution curve (**D**) of magnesium oxide nanoparticles (MgONPs).

The micrographs revealed that the MgONPs were largely spherical with a relatively uniform diameter of approximately 50 nm (Figure 1A,B), and they were distributed in the matrix extraction solution and deionized water with similar irregular but spherical particle-like shapes (Figure S1). The mean zeta potential of the MgONPs in the matrix extraction solution and the matrix extraction solution was −20.05 and −24.85, respectively, revealing the dispersion stable of MgONPs (Table S1). In particular, the nitrogen adsorption–desorption isotherms and corresponding pore size distributions are roughly shown in Figure 1C,D. Pore size analysis for adsorption clearly demonstrated that the MgONPs possessed mesopores with average diameters of 25.0 and 50.0 nm (Figure 1D). An analogous investigation indicated that the NPs of Figure 1C should be regarded as the textural mesopores. In addition, the data of the strong adsorption at P/P0 of approximately 1.0 should reflect the large mesoporous cavities of MgONPs [17].

### 3.2. Seed Germination

As shown in Figure S2, the MgONPs had no significant effects on the tobacco seedling germination rate. Marusenko et al. reported that nanoscale TiO_2_ and ZnO particles do not affect tomato seed germination in the range of concentrations studied (0–750 μg/mL) [7]. Another study revealed that nano-TiO_2_ can promote the germination of spinach seeds and increase seedling vigour and growth, which can trigger the antioxidative mechanism [18]. These differences depended on the type of nanoparticle materials, plant species and plant substrates, such as the type of soil and the culture medium, involved [4]. Similar to other NPs [19], MgONPs can also produce large aggregates in aqueous media, which probably decreased the negative effect on biological organisms and did not result in apparent toxicity. Although there was no prominent increase in the germination rate when the plants were exposed to 250 μg/mL MgONPs, the seeds did not exhibit any toxicological effects at this concentration. Interestingly, this concentration could kill *R. solanacearum* [13], revealing that the MgONPs could potentially be used in the future to protect plants. However, before large-scale application, a thorough evaluation of the interaction between MgONPs and plants is necessary.

### 3.3. Effect of MgNPs on Tobacco Growth and Chlorophyll Content

**Figure 2:**
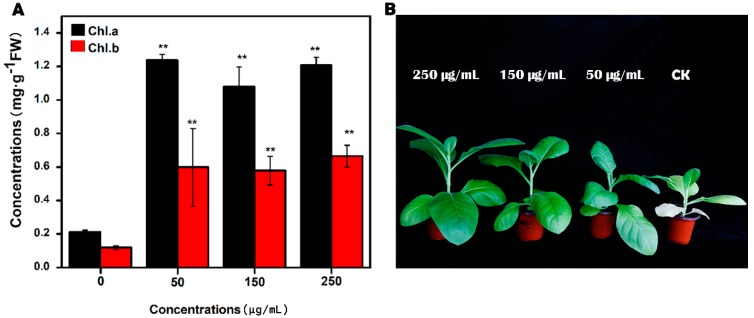
Effect of the different concentrations of MgONPs on the chlorophyll analysis of the tobacco plant, (**A**) chlorophyll concentrations, and (**B**) the phenotype of tobacco plants with MgONPs of 250, 150, 50 and 0 μg/mL from the left to right. Error bars represent the standard deviation, ** *p* < 0.01.

As shown in Figure 2, when treated with concentrations ranging from 0 μg/mL to 250 μg/mL, the MgONPs increased the chlorophyll a and b contents remarkably from 0.21 and 0.12 μg/g to 1.21 and 0.67 μg/g, respectively, after 30 days of treatment. Consistent with these results, Jhansi et al. also indicated that peanut seeds soaked in a 500 μg/mL MgONP (particle sizes ranging from 10 nm to 80 nm) suspension for 12 h can enhance the chlorophyll content in plant leaves [20,21]. The chlorophyll contents of the plants are considered a critical parameter to analyse phytotoxicity [20]. They were used as an important indicator of the photosynthetic performance of plants, because of its pivotal position in the progress of the assimilation system of plants. In addition, their content changed with maturity, age, daytime length, illumination intensity and the environment [21].

In addition, Magnesium is a central element in all types of chlorophyll and is thus crucial to photosynthesis in plants. Chlorophyll cannot capture sun energy needed for photosynthesis without magnesium. Magnesium also has very significant implications in partitioning, utilization of photo-assimilates, photophosphorylation (including ATP formation) and photo-oxidation in leaf tissues and the generation of reactive oxygen species [22,23]. Overall, Mg likely acts in the chlorophyll structure, which might be the primary reason for the improved response of the plants treated with MgONPs.

Similarly, Juhel et al. clearly demonstrated that the light reactions of photosynthesis obviously increased following treatment with nanoalumina, thereby enhancing biomass accumulation in *Lemna minor*; thus, the metal NPs are related to enhanced energy transfer efficiency of the isolated reaction centres [24]. However, few reports supported this hypothesis that nanomaterials interact with photosystems [25]. In addition, given their high surface reactivity, NPs can enlarge and create root pores, leading to high hydromineral transportation in the roots [26].

In this study, we hypothesized that the addition of low concentrations of MgONPs could induce a synergistic promotional effect on the growth rates of the tobacco plants and possibly enhance the photosynthetic performance of the plants. In addition, exposure to MgONPs may lead to a dramatically high increase in the Mg content. To maintain healthy growth and the green leaf phenomena involved in the resistance to adversity, such as antioxidant systems and photosynthesis in plants, we investigated the effects by which MgONPs activated the plant antioxidant systems through additional experiments.

### 3.4. Evaluation of the Antioxidant Enzyme Activities

**Figure 3:**
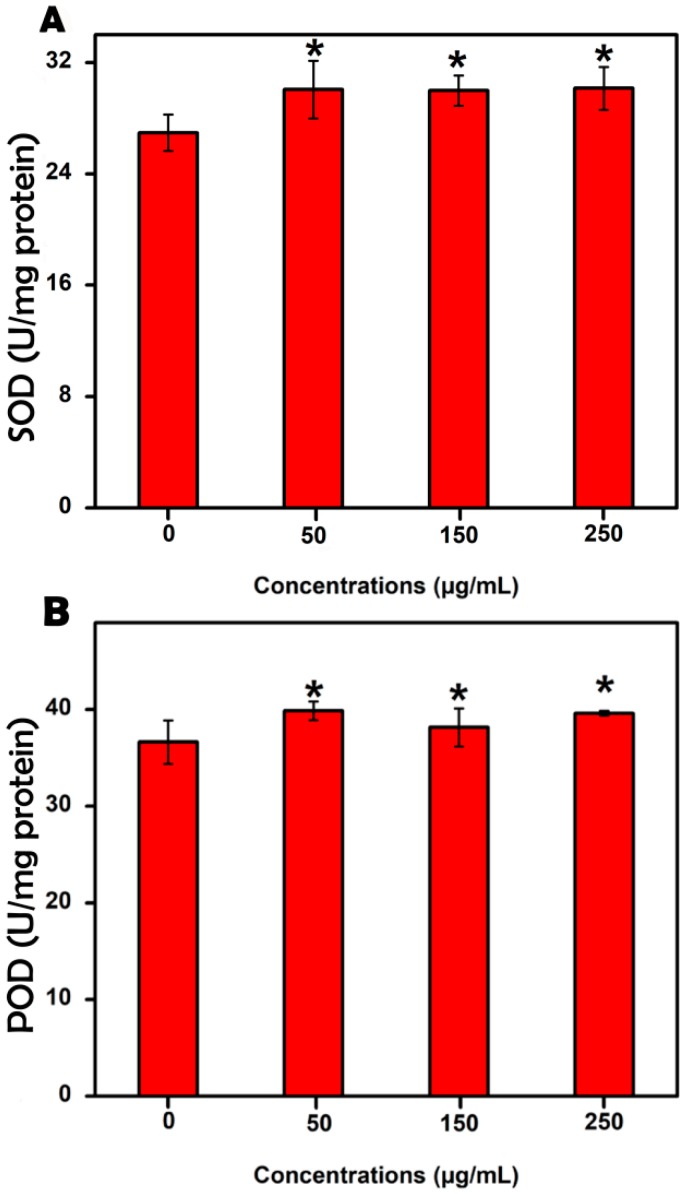
Effect of the MgONPs on the antioxidant system, (**A**) SOD activities, and (**B**) POD activities. Error bars represent the standard deviation, * *p* < 0.05.

As shown in Figure 3, marked differences were found between the MgONP treatment and control groups with respect to their antioxidant system, including SOD and POD activities. Specifically, the SOD and POD activities following exposure to 250 μg/mL MgONPs were 30.15 and 39.63 U mg^−1^ protein, respectively, which were significantly higher than those of the control group (26.95 and 36.63 U mg^−1^ protein, respectively) and much higher than those of the 50 and 150 μg/mL groups. Therefore, 250 μg/mL MgONPs enhanced the activity of the antioxidant enzymes.

To grow and develop effectively, plants have a variety of defence mechanisms against external stress, and the antioxidant system is one of the mechanisms that plants use to resist damage caused by their own reactive oxygen free radicals [27]. Previous studies also noted that plant exposure to engineered NPs, such those containing Ag, are becoming a topic of increasing concern due to the over-accumulation of reactive oxygen species (ROS), which attack biofilms, lead to lipid peroxidation and induce DNA and protein damage [28]. Therefore, ROS are used as markers to predict the toxicity of NPs [29]. Notably, the chloroplast is not only an important organelle for photosynthesis but also the site that produces ROS [30].

In general, once external stress stimulation leads to the destruction of the balance mechanism, changes in some enzyme activities directly reflect the degree of stress on the plant cells. Numerous studies suggest that SOD and POD are antioxidant protective enzymes that alleviate part of the oxidative stress and maintain the dynamic balance of ROS in the organism. By contrast, SOD can scavenge superoxide ion free radicals during the course of the superseding of the organism, stabilize the membrane structure and prevent ROS formation, whereas POD primarily catalyses the decomposition of hydrogen peroxide to water and oxygen [31]. In addition, treatment with MgONPs resulted in high POD activities in tobacco plants; POD could remove excessive H_2_O_2_ in the cells, reduce the peroxidation effect of H_2_O_2_ on membrane lipids and stabilize the membrane structure [31]. These effects may influence the stimulation of POD activity and alleviate oxidant stress [32].

### 3.5. Malondialdehyde, Relative Water and Protein Content Detection

Despite the dosing intensity and differences in the MgONP concentrations, the MDA content of the tobacco plants was no significant difference among these treatments compared with the controls. Figure S3 indicates that the MDA levels in different MgONP concentrations (250, 150, 50 and 0 μg/mL) were 3.34, 3.43, 3.52 and 3.54 nmol/mg protein, respectively. No discernible differences were found among all of the treatments.

Environmental stress can lead to the production of excessive free radicals in plants, which attack the lipid membrane system, result in lipid membrane peroxidation and lead to intracellular electrolyte leakage [33]. When exposed to an adverse environment or unfavourable stress, the MDA content of plants is one of the primary products in scavenging free radicals, which can change the structure and function of the cell membrane to lead to cell destruction; the MDA content is an indicator of lipid peroxidation levels, and high MDA levels result in serious damage to the cell membrane system [34]. The MDA content is widely used to evaluate oxidative stress [35].

Our results were consistent with previous studies on the MDA levels of other plants, such as the TiO_2_ nanocomposites of surface modification towards *Vicia faba* [36]. By contrast, Wang investigated the effects of CuONP and CuNP in rice seedlings; they found a significant increase in the MDA levels that was directly proportional to their concentration after cultivation with such nanomaterials [37]. Large quantities of engineered NPs have been proven to display the toxic effects of metal NPs caused by the increase in the lipid peroxidation product MDA [38]. However, not all plants demonstrate positive effects or even any toxicity following exposure to or treatment with NPs. Similarly, one cannot attest that no effects may occur [39].

Subsequently, we exposed these samples to various concentrations of MgONPs and studied the relative water content, which is an indicator of crop water status [15]. The relative water content of the MgONP-treated seedlings did not significantly differ. Exposure of the seedlings to 50, 150 and 250 μg/mL NPs led to water content levels of 79.63%, 81.90% and 83.45%, respectively, whereas the relative water content of the control was 84.11% (Figure S4). This type of minimal influence was also found in chickpeas treated with 1.5 ppm nano-ZnO [15]. Some studies have documented that the plant water status is an indicator to evaluate heavy metal toxicity [40]. Alternatively, no significant correlation was found between plant water content and biomass yield and phytomedicine contents in plants [40]. Therefore, our data were used to gauge the non-negative effects of MgONPs on plant physiology.

In addition to the relative water content, the protein content was used to analyse the effects of MgONP treatments. Similar performance in the content of protein (Figure S4) in the case of NP-treated and control plants was noted. Thus, exposure to NPs had no significant influence on the protein content of tobacco plants. Another study also found that the protein concentrations in bean plants have no direct relation to Mg deficiency [41].

Usually, toxic materials will reduce plant metabolic activity [42] and further induce reactive oxygen free radicals (ROS) production in the plant cells. After producing ROS, the double bonds on the fatty acids of the membrane phospholipids are oxidized and damage the membranes, leading to the inhibition of plant growth and potential death. Therefore, considering these physiological characteristics of plant sensitivity, phytotoxicity evaluation may be used to directly detect the toxic effects of abiotic and biotic environmental stress [43]. Collectively, the MgONPs could improve plant growth via positive physiological impacts, such as increased enzymatic defences, but did not cause direct injury or stress.

### 3.6. Morphological and Anatomical Analyses

To evaluate the structural changes in the tobacco plants after incubation with MgONPs, the root, stem and leaves were cut with minimal damage to the samples to prepare standard paraffin sections. Specifically, in view of the high concentration treatment showing a statistically significant difference compared with the control, we selected 250 µg/mL MgONPs as the treatment concentration.

**Figure 4:**
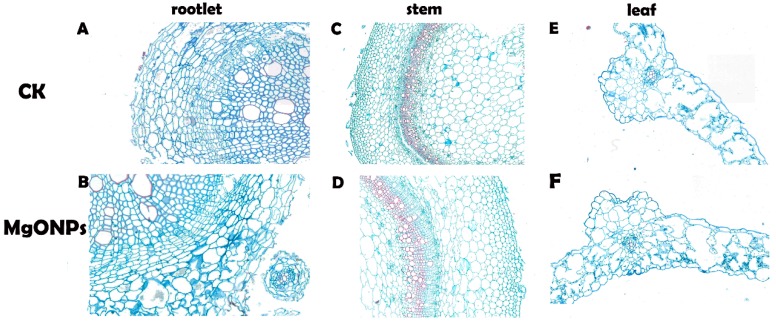
Paraffin section images (cross-sections) of the tobacco roots (**A**, **B**), stems (**C**, **D**) and leaves (**E**, **F**) exposed to 250 μg/mL of MgONPs and distilled water.

Our observations using light microscopy clearly showed that the plants exposed to 250 µg/mL MgONPs showed little or no alterations in the anatomy and morphology of the tobacco plants when compared with the control group (Figure 4). In the present study, the root, stem and leaves of the MgONP-treated and untreated plants grew well with compact structures, including the cortex, epidermis and pericycle. In the stems, the xylem ring size and shape did not vary significantly between the MgONP treatments and control. In addition, the intercellular spaces were unaltered. These results confirmed that 250 µg/mL MgONPs did not induce negative effects on the tobacco plant.

**Figure 5:**
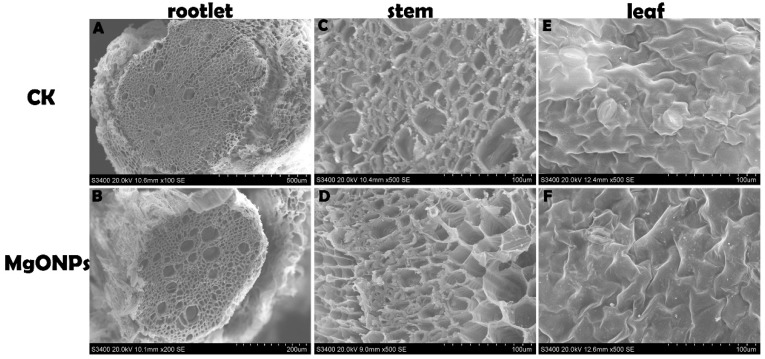
Morphological interactions with or without MgONPs treatment on 30 days using SEM analysis. (**A**, **B**) rootlet, (**C**, **D**) stem, (**E**, **F**) leaves.

In addition, SEM investigations indicated that the plants of both treatment groups did not induce any obvious alterations in the stem, roots and leaves (Figure 5). The plants exposed to MgONPs showed no adverse effects, such as distortion, curling, chlorosis or necrosis in the leaves. This finding was consistent with the results of Krishnaraj et al. [25] who confirmed that 10 ppm of AgNPs treatment caused little or no change in the morphology of *Bacopa monnieri*. By contrast, interaction with silver ions (0.1 mM–1 mM) changed the biochemical, physiological and morphometric parameters, such as distorting the root epidermal structure or altering the anatomical features of sunflower plants [44]. ZnONPs (1000 mg/L) and copperNPs (500 mg/L) have also been demonstrated to slow the growth of seedlings [45,46]. In this type of study, an evaluation of the direct and indirect effects on the plants should be based on different nanomaterials and various plant species [4]. In addition, plants treated with low concentrations of AgNPs grew better than the control, whereas numerous alterations in the cell morphology and structural characteristics were observed following exposure to high AgNP concentrations [4]. Therefore, the advantage of MgONPs over other nanomaterials was that the plants did not exhibit any significant injury and stress following 250 µg/mL MgONP exposure, which indicated that the plants were likely to possess enzymatic defences to tolerate relatively low MgONP concentrations. Once adsorbed to the root surface, the nanomaterials were likely to interrupt the absorption of macro- and micronutrients of the plants and reduce nutrient uptake, thereby decreasing the chlorophyll content [20]. Recently, our detailed study in which tobacco plants were exposed to MgONPs through root irrigation confirmed that MgONPs had outstanding advantages, such as a lack of aggregation of the NPs on the root surface.

### 3.7. Uptake and Translocation of MgONPs

**Figure 6:**
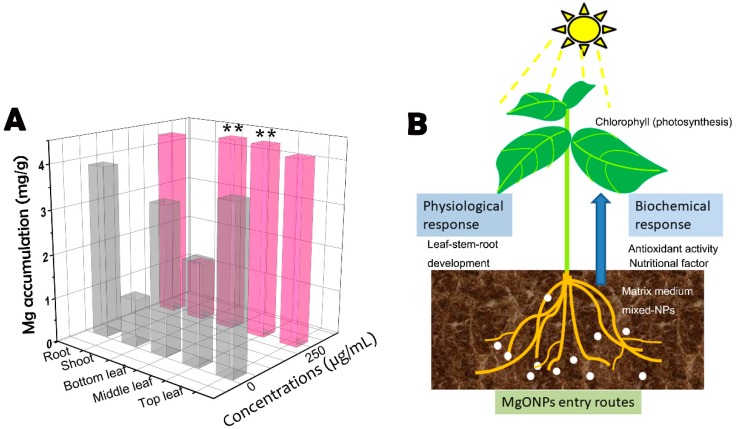
Mobilization and accumulation of Mg among the tobacco plants was recovered by ICP-MS (**A**), and a schematic representation of the uses and dispersion of MgONPs to interact with the plants (**B**). Accumulation of the Mg metal ion in the roots, shoots and leaves (bottom, middle and top leaves) of treated or untreated plants by 250 μg/mL MgONPs, ** *p* < 0.01.

ICP-OES is the most frequently used detection technique to locate the metals in plant tissues [4]. In this study, all the 30 day plant samples were harvested, and the magnesium concentrations were examined in the leaves, stems and roots via ICP-OES. The concentrations of elemental magnesium were normalized to the control plants using the dried mass of the plant samples, and the data were processed to show the percentage of magnesium recovered in each plant section. The magnesium contents in the dried tobacco plant tissues are shown in Figure 6A. The data of the control group (Figure 6) indicated that 9.30 mg/g magnesium particles accumulated in the leaves, including the bottom, middle and top leaves, followed by 3.88 mg/g recovered in the root sections and 1.06 mg/g in the stems. However, the corresponding data following exposure to MgONPs were 12.93, 4.27 and 1.34 mg/g. Compared with the results of the two treatments, more magnesium accumulated in the tobacco plant tissues, which were treated with 250 μg/mL MgONPs and significantly increased in the lower and middle leaves. In this study, the ICP-OES data demonstrated that the magnesium could easily penetrate into the roots and pass through the shoots, reach the leaves, and deposit on the lower and middle leaves and become biodistributed throughout the plant by its vascular system. However, CuONP was found to be translocated from the roots to the shoots in the plant [47]. NPs taken up by the root cells in soil are transported in unidirectional pathways by the xylem simultaneously with water. This biotransformation first travels through the humic acid and root exudates, followed by absorption by the surface pores of the roots [39]. Given the high surface reactivity, NPs can create new root pores and increase hydromineral and nutrient uptake [16]. But, NPs in plant systems cannot easily translocate both the metal ions and metal oxides into the plant stems, especially for large particles, which have difficulty penetrating plant tissues [16]. In addition, some metals penetrate from the root to the stems, and only a small percentage reaches the leaves [4,48]. The trichomes (epidermal hairs) on the upper epidermis of the plant leaves also limit the transport of nanoparticle on the leaf surface [16]. Therefore, our experimental treatments conducted by root irrigation probably also improved the transportation of MgONPs and magnesium ions.

Notably, several reports demonstrated that charged NPs can be targeted to specific locations of the plant tissues for delivery to form aggregates in the targeted positions [49]. To the best of our knowledge, MgONPs are negatively charged in the extraction solution. Thus, these interactions may be one crucially enhanced pathway to accelerate absorption. Although all the plants could transport nutrients, tobacco plants have tremendous potential to accumulate numerous metals (NPs and ions) because of the thin long roots that result in increased surface area. In summary, our experiments confirmed that elemental magnesium was predominantly taken up and biotransformed in the plants (Figure 6B). However, there is a lack of detailed investigations on the pathways responsible for uptake and biotransformation.

In the range of concentrations (50–250 μg/mL) of the NPs tested, they either slightly or prominently enhanced tobacco growth and development. Once the elemental magnesium was taken up by the tobacco plants, they functioned as an essential mineral element. The trends observed were primarily due to the chlorophyll content in the plant and enhanced light absorption of the mineral elements [7]. Magnesium is an essential micronutrient, and, eventually, MgONPs could be used to overcome Mg deficiency in plants in agricultural production. As observed by the abnormal decrease in the MDA content, MgONPs could relieve the stress of the lack of magnesium on the plant cell membrane of the control, which is consistent with the results of a previous study [35]. However, further study about the influence of MgONPs on other elements would have an enabled an assessment of potential shifts in other important metabolic processes as a function of exposure. In addition, using proteomic, genomic and metabolomic approaches will be needed to better understand the mechanisms underlying the interactions of the NPs with plants that are involved in a number of metabolic and physiological activities [39].

## 4. Conclusions

Little is known about the nanoparticle–plant interactions when exposed to agricultural and environmental systems. In this study, we demonstrated that the accumulation of Mg in the tissues of the root, stem, and leaves at the concentrations tested was the causal factor to enhance the chlorophyll content, antioxidant system, biomass and plant growth. Thus, Mg accumulation could have potential applications in agriculture, such as controlling plant diseases or supplementing nutrients in the future. Our results indicated no inhibition on seed germination. Once the elemental Mg were taken up by the tobacco plants, they were probably biodistributed throughout the whole plant by the vascular network. After exposure to MgONPs in the matrix for 30 days, the growth of the tobacco seedlings was significantly enhanced. Increased POD and SOD activities and unchanged MDA and protein contents coupled with the relatively consistent water levels may indicate that the NPs increased the oxidative stress of the plants and did not damage the membranes of the tobacco plants. Notably, MgONPs were confirmed to serve as an outstanding Mg supplement for plants. Therefore, the NPs had a twin role of enhancing the essential nutrient uptake of Mg and enabling its use as a cofactor to mobilize nutrient enzymes. Overall, MgONPs at the concentration level tested could be potentially valuable in agricultural practices to facilitate the physiology and biochemistry of plants and provide higher benefits for a targeted agro-economic trait.

## Supplementary Materials

The Supplementary Materials are available online.
