# Supplementary material for: MgONPs Can Boost Plant Growth: Evidence from Increased Seedling Growth, Morpho-Physiological Activities, and Mg Uptake in Tobacco (Nicotiana tabacum L.)
Source: Molecules. 2018 Dec 19;23(12):3375. doi: 10.3390/molecules23123375 (PMC6321585; doi:10.3390/molecules23123375)
Supplement: Supplementary file 1 [file molecules-23-03375-s001.pdf]

# Supplementary Material

## MgONPs Can Boost Plant Growth: Evidence from Increased Seedling Growth, Morpho-Physiological Activities, and Mg Uptake in Tobacco (*Nicotiana tabacum* L.)

**Lin Cai [1,†], Minghong Liu [2,†], Zhongwei Liu [3,†], Huikuan Yang [1], Xianchao Sun [1], Juanni Chen [1], Shunyu Xiang [1] and Wei Ding [1,*]**

[1] College of Plant Protection, Southwest University, Chongqing 400715, China; lincai0203@163.com (L.C.); kuan320914@163.com (H.Y.); sunxianchao@163.com (X.S.) chenhuanni521@126.com (J.C.); xiangshunyu0325@163.com (S.X.)
[2] Zunyi Branch Company, Guizhou Tobacco Company, Zunyi 563000, China; lmh859@163.com
[3] Guizhou Key Lab of Agro-Bioengineering, Guizhou University, Guiyang 550025, China; zwliu@gzu.edu.cn
* Correspondence: dwing818@163.com
† The authors contributed equally to this work

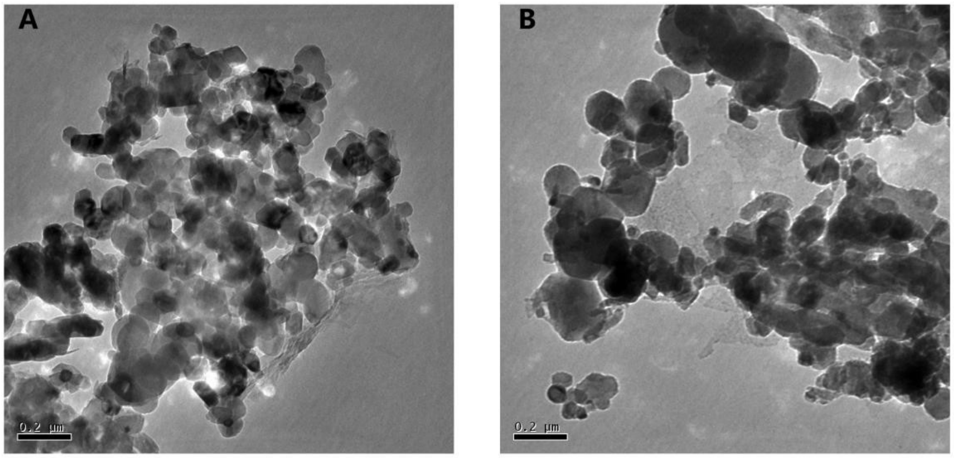


**Figure S1.** TEM Morphological and dispersibility characterization of MgONPs. (A) MgONPs dispersed in the deionized water and (B) in the matric extractiong solution at pH 7.0.

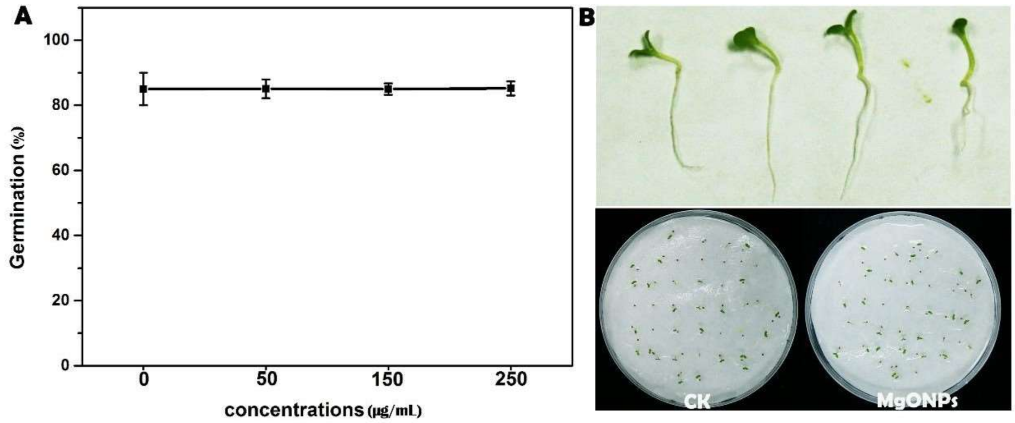


**Figure S2**. Germination of tobacco seeds after treatment with different concentrations (0, 50, 150, and 250 μg/mL ) of MgONPs for 5 days. (A) Germination rate, (B) root length and stem length of the seedlings and (C) phenotypes of 5-day-old tobacco seedlings grown on media with water. Error bars represent the standard deviation, * and ** indicate $p < 0.05$ and $p < 0.01$, respectively.

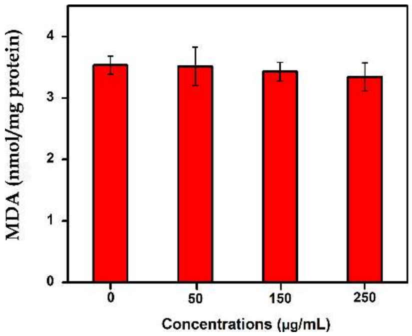


**Figure S3**. Effect of the different concentrations of MgONPs on the malondialdehyde (MDA) content of tobacco plants. Error bars represent the standard deviation, * and ** indicate $p < 0.05$ and $p < 0.01$, respectively.

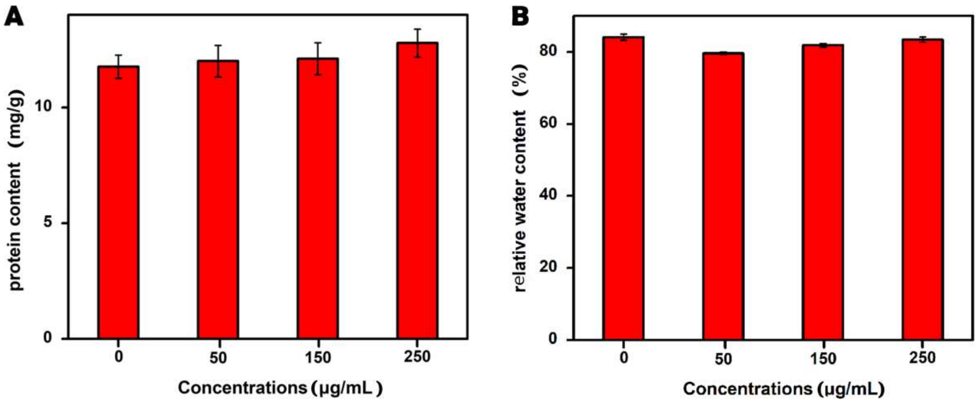


**Figure S4**. Effect of MgONPs on the protein and relative water content of tobacco plants. The trend shows none significant effect between each treatments.

Table S1 Zeta potential analyses

| liquid | Zeta potential (mV) |
| --- | --- |
| MgONPs in the matrix extraction | -20.5±2.6 |
| matrix extraction | -24.7±3.4 |
